# Breakfast Habits and Associations with Fruit and Vegetable Intake, Physical Activity, Sedentary Time, and Screen Time among Swedish 13–14-Year-Old Girls and Boys

**DOI:** 10.3390/nu13124467

**Published:** 2021-12-14

**Authors:** Björg Helgadóttir, Hanna Baurén, Karin Kjellenberg, Örjan Ekblom, Gisela Nyberg

**Affiliations:** 1 The Swedish School of Sport and Health Sciences (GIH), 114 86 Stockholm, Sweden; karin.kjellenberg@gih.se (K.K.); orjan.ekblom@gih.se (Ö.E.); gisela.nyberg@gih.se (G.N.); 2 Karolinska Institutet, Department of Clinical Neuroscience, Division of Insurance Medicine, 171 77 Stockholm, Sweden; 3 Karolinska Institutet, Department of Biosciences and Nutrition, 171 77 Stockholm, Sweden; hanna.bauren@stud.ki.se; 4 Karolinska Institutet, Department of Global Public Health, 171 77 Stockholm, Sweden

**Keywords:** breakfast habits, nutrition, adolescents, accelerometry, moderate-to-vigorous physical activity, screen-time

## Abstract

This study explored whether breakfast habits were associated with intake of fruits and vegetables, minutes in moderate-to-vigorous physical activity (MVPA), minutes spent sedentary, and screen time among adolescents. Cross-sectional data were collected among 13–14-year-old boys and girls (*n* = 1139). Breakfast habits and screen time were determined via questionnaire, fruit and vegetable intake were determined through dietary recall, and physical activity and sedentary time were determined via accelerometers. Multilevel mixed models and general estimation equation models were applied. Almost 40% of participants skipped breakfast at least one day of the week. Participants with irregular breakfast habits on weekdays had lower fruit and vegetable consumption by 26.7 g (95% CI = −49.3, −5.9) while irregular breakfast habits during the whole week were associated with higher levels of screen time (OR = 1.5, 95% CI = 1.1, 2.1) compared to regular breakfast habits. Girls with irregular breakfast habits on weekdays had 7.7 min more sedentary time (95% CI = 0.8, 15.7) than girls with regular breakfast habits, while the opposite was found in boys (β = −13.3, 95% CI = −25.3, −2.6)). No significant associations were found for MVPA. Regular breakfast habits should be encouraged, as they might contribute to a higher intake of fruit and vegetables and are associated with lower levels of screen time, although further studies are necessary to establish causation.

## 1. Introduction

Irregular meal patterns, i.e., skipping breakfast and/or lunch, have been associated with an unhealthy diet in general, primarily through more snacking between meals [1]. In adults, leisure-time physical activity, structured exercise, and some aspects of dietary habits, such as candy/cake and alcohol consumption, have been shown to predict future sedentary behavior [2]. Such studies are lacking in adolescents. As health habits tend to cluster [3], it is of interest to investigate whether breakfast habits are associated with other health habits such as diet, physical activity, sedentary time, and screen time, especially since such habits have been associated with a higher risk of obesity and morbidity in adulthood [4,5,6].

A sufficient diet is particularly important for children and adolescents since they are growing rapidly and require a high amount of nutrients, such as vitamins and minerals, which are abundant in fruit and vegetables [7]. In Sweden, the recommended intake is 500 g of fruit and vegetables per day for children from 10 years old [8]. In a national dietary survey of adolescents aged 11–17 years from 2016–2017, only 7.5% of the participants consumed 500 g or more of fruit and vegetables per day, and the mean intake was 233 g per day, with girls consuming more than boys [9]. Irregular breakfast has been related to lower intake of fruit and vegetables among 11–15-year-old children [10,11] in studies using general food frequency questionnaires. There is a lack of studies based on diet registration, which gives a more precise measure of the quantity of intake, as well as studies stratifying their analyses by gender.

Another important health habit is physical activity, where the recommendation in Sweden, as well as by the World Health Organization is 60 min of daily moderate-to-vigorous physical activity (MVPA) for children and adolescents [5,12]. Today, only 43% of boys and 23% of girls in Sweden reach the recommendation [13]. Studies that have investigated breakfast habits in relation to physical activity are scarce, as more focus has been on linking breakfast habits with overweight and obesity. 

Studies on the association between breakfast skipping and device-based measured sedentary time are infrequent. Instead, the focus is more often on screen time as a subset of sedentary time. Screen time has increased among Swedish 15-year-olds between 2009 and 2014 [14]. No official Swedish guidelines exist for screen time, but the Canadian guidelines state that screen time should be limited to no more than two hours per day, outside of school activities [15]. Previous studies [16,17] have linked breakfast consumption and sedentary behavior, based on self-reports. However, these studies do not take into account that screen time and breakfast habits might differ between weekdays and weekend days. Since both food intake and sedentary behavior are notoriously difficult to report accurately, the use of recall self-reports in studies of sedentary behaviour and food intake is most probably the reason for conflicting results.

The evidence for an association between breakfast and both fruit and vegetable intake and amount of physical activity and sedentary time, especially device measured, are limited and inconclusive. Gender differences have been found in previous studies regarding physical activity patterns [13], fruit and vegetable intake [9], and breakfast habits [18]; therefore, stratification by gender is advisable. Therefore, the primary aim of this study was to explore whether breakfast habits among 13 to 14-year-old adolescents were associated with the intake of fruits and vegetables, physical activity patterns (minutes in moderate-to-vigorous physical activity (MVPA) and minutes spent sedentary), and screen time. A secondary aim was to investigate any gender differences in the associations.

## 2. Materials and Methods

### 2.1. Study Design, Setting, and Participants

The data in this study were collected from the cross-sectional study “Physical activity for healthy brain functions in school youth”. Schools within a two to three-hour driving distance from Stockholm were invited to participate. Schools with a sports profile, small schools with classes with less than 15 students, and language preparation schools were excluded. A total of 558 schools were invited to participate, and 84 accepted. Of those, 40 schools were chosen for inclusion in the study based on a variation in the type of municipality and the socioeconomic background of the schools. However, six dropped out, leaving a total number of 34 schools. At each school, students attending 1–4 classes in the 7th grade were invited to be a part of the study (*n* = 1556 students), thus resulting in 1139 students participating in the data collection (participation rate of 73%). More information on the study is available in Nyberg et al. (2021) [19]. 

### 2.2. Ethical Statement

The study was approved by the Swedish Ethical Review Authority (dnr: 2019-03579) and was conducted in accordance with the Declaration of Helsinki. Students and their parents or guardians received an informational letter about the study and gave written informed consent. Participants received a gift card of 300 sek (approximately $30) for their participation.

### 2.3. Data Collection

Data collection took place from September to December 2019. The students, along with supervising teachers, were invited in small groups (up to 60 at one time) to come to the study center at the Swedish School of Sport and Health Sciences. Each group spent approximately 4–5 h on various data collection activities, from morning (usually around 8:00) until lunch. The participants were provided with a small breakfast at the start of the day as well as lunch after the data collection was finished. 

The participants were asked to answer a questionnaire and could ask for assistance and clarifications from the researchers or their teachers. Participants were instructed on how to use the web-based dietary assessment tool [20]. Anthropometric measurements were taken, and physical activity data were collected with the help of accelerometers. The parents (or guardians) of the participants were also asked to answer a short online questionnaire. The measures are described in greater detail below.

#### 2.3.1. Breakfast Habits

Breakfast habits on weekdays were measured with the question “How often do you eat breakfast (more than a glass of milk or juice) on weekdays?” The response options ranged from “5 weekdays” to “I never have breakfast on weekdays”. The same question for the weekend had the response options: “I usually have breakfast both days of the weekend”, “I usually have breakfast one of the days on the weekend”, or “I never eat breakfast on the weekend”. Each variable was dichotomized into regular breakfast (never skipping breakfast) and irregular breakfast (skipping breakfast at least once). Furthermore, a third breakfast variable for the whole week was created, which was dichotomized into those that had regular breakfast (never skipped breakfast on weekdays and weekends) and irregular breakfast (skipped breakfast at least once during either weekdays or weekends). 

#### 2.3.2. Intake of Fruit and Vegetables

Data on the participants’ diets were registered via RiksmatenFlex, a web-based dietary assessment tool created by the Swedish Food Agency [21], using a repeated 24 h multiple pass recall. RiksmatenFlex is self-administrative and can be used on smartphones, tablets, and computers. In the RiksmatenFlex program, the participant enters the meals they have consumed and receives follow-up questions about the amount and specific food type. To help to determine serving sizes, pictures of different amounts of the dish in question were shown, and the participant chose the amount most similar to their portion size. This method gives detailed information about nutrient content and specific food groups in the registered diet and has been validated in this age group [20]. 

After an introduction to the program, the participants were able to register their diet themselves. The participants registered their diet for three whole days, i.e., the day that they visited the research center, the preceding day, and a randomly selected day after. Therefore, both the preceding day and the randomly selected day were registered retrospectively. The data from the day at the research center were excluded as the participants’ diets were provided for them for part of the day and therefore did not reflect a normal day. Therefore, the diet data were an average of the other two days. The participants registered the day that preceded the visit at the research center with guidance from the researchers and the last day they registered at home by themselves. The registration was checked for quality and completeness [21]. A total of 1036 participants had two days of dietary registrations, while 97 had one day and 6 participants had no completed registered days. Of those that had two complete days, 317 had two weekdays (Monday to Thursday), while the remaining participants had one weekday and one weekend day (Friday to Sunday). 

The fruit and vegetable intake was based on the intake of vegetables, legumes, root vegetables, mushrooms, fruits, and berries in grams. Components of dishes, such as the tomatoes included in a lasagna were counted as well. The intake in grams was summed up and averaged over the two days. Additionally, energy intake in kilocalories (kcal) was calculated and averaged over the included days. 

Participants were classified into under, plausible, or over-energy reporters using the ratio between energy intake (EI) and total energy expenditure (TEE). The calculations for TEE were done by using data on physical activity, weight (kg), and gender, as previously described [22]. Plausible reporters were categorized as those falling within one standard deviation (0.31) around the mean (0.82) of the ratio, while those that had a ratio of less than 0.51 were classified as under-reporters and those with a ratio of more than 1.13 were classified as over-reporters. Only those with two days of registered dietary intake were included in the calculations. A total of 1008 participants fulfilled the criteria. Of those, 690 (68.5%) were classified as plausible reporters. 

#### 2.3.3. Physical Activity 

Data about physical activity were collected via triaxial accelerometers (model GT3X+, Actigraph, LCC, Pensacola, FL, USA). The participants received the accelerometers with written instructions and received an introduction by trained staff. Participants were instructed to wear the accelerometer at all times when awake, on their right hip, during the seven measuring days, except for activities in water (e.g., showering, swimming). The monitors collected activity data at a sampling rate of 30 Hertz and were down-sampled into 5 s time intervals for analysis, using ActiLife version 6.13.3. Non-wear time was removed, which was defined as 60 consecutive minutes or more of zero counts with zero-minute spike tolerance using triaxial data. A day was considered valid when 500 min of data or more remained after removing non-wear time. For the assessment to be valid, at least three valid days of measurement were required, of which at least one was a weekend day (*n* = 903). Uniaxial counts from the vertical axis of the accelerometer data were categorized into minutes spent in sedentary intensity (0–100 counts/minute) and moderate-to-vigorous physical activity (MVPA, ≥2296 counts per minute) [23]. Additionally, the wear time in minutes was calculated and averaged across the included days. 

#### 2.3.4. Screen Time 

Screen time was measured via questionnaire including the questions: “During a normal weekday, outside of school, how much time do you spend looking at a screen?” and “During a normal weekend, how much time do you spend looking at a screen?”. The response alternatives were no time at all, less than 1 h, 1–2 h, 3–4 h, 5–6 h, and 7 h or more. These were dichotomized into 2 h or less and more than 2 h in accordance with recommendations for screen time for this age group for weekdays and weekends. 

#### 2.3.5. Background Characteristics

Gender and country of birth were reported via questionnaires by the participants. There was a separate questionnaire for the parents/guardians of the child, where the information about parental/guardian education level was collected. The highest education reported was used to indicate the socioeconomic status of the household, which was dichotomized into 12 years or less or more than 12 years. The municipality where each school was located was categorized as urban or rural using the Swedish municipality classification 2017 [24].

#### 2.3.6. Anthropometrics

Weight and height were measured by standardized, objective procedures using a calibrated scale (Tanita BC-418, Tanita Corporation, Tokyo, Japan) to the nearest 0.1 kg and a stadiometer (SECA 5123, SECA Weighing and Measuring Systems) to the nearest mm. Body Mass Index (BMI) was calculated as body mass (kg) divided by height (m) squared. Then, the BMI was categorized according to sex- and age-specific values from the International Obesity Task Force (IOTF) 2012 [25].

### 2.4. Statistical Analyses

Descriptive statistics were presented as means with standard deviations or percentages. Differences between girls and boys for background variables were tested with chi-square tests for categorical variables and independent *t*-tests for continuous variables. The associations between breakfast habits and the continuous outcome variables (intake of fruit and vegetables, sedentary time, MVPA) were tested with multilevel mixed linear regression models. A random intercept was modeled for each school. For the dichotomous outcomes (screen time), multilevel general estimation equation models were used. We have applied the models with two levels: the school level and the individual/student level. We checked the model fitness and heteroscedasticity by conducting goodness-of-fit tests, plotting residuals as well as their relationships with predictors. Therefore, the bootstrapping estimation was performed to produce sensible estimates for standard errors in the multilevel model if the suboptimal fitness of the model or heteroscedasticity was detected. The analyses of screen time as outcome were adjusted for gender, parental education, and BMI. The models with fruit and vegetable intake as outcome were additionally adjusted by energy intake, while the analyses for MVPA and sedentary behavior were additionally adjusted for accelerometer wear time. The analyses were done in three steps for all outcomes: (1) All participants were included and the models were adjusted for the confounders described above. (2) All models were stratified by gender and adjusted for the same confounders (except gender). (3) Interaction terms gender*breakfast were added to the models described in step 1. Results from step 3 are only reported in text when significant. Sensitivity analyses were performed for the analyses on fruit and vegetable intake, using only plausible energy reporters. The level of statistical significance was set at *p* < 0.05. The data analyses were performed in IBM SPSS Statistics, version 27 (IBM Corp., Armonk, NY, USA).

## 3. Results

The sample was quite evenly divided between girls (51%) and boys (49%), as shown in Table 1. The mean age of the participants was 13.4 years (SD = 0.3) with the majority having at least one parent with more than 12 years of education (72%). Approximately 14% of the participants were born outside of Sweden. Almost 40% of the participants skipped breakfast at least once during the week. Boys were more likely to eat regular breakfast on weekdays and during the whole week compared with girls (*p* = 0.002 and *p* = 0.013, respectively). A higher proportion of girls reported ≥2 h of screen time during weekends compared with boys (*p* < 0.001), and girls also had more minutes spent sedentary and fewer minutes in MVPA than boys (*p* = 0.001 and *p* < 0.001, respectively). 

Those who had irregular breakfast habits on weekdays were found to consume less fruit and vegetables by 26.7 g (95% CI = −49.3, −5.9) compared with those who had regular breakfast habits (Table 2). After stratifying by gender, the association remained true and strengthened for girls but not for boys, although the interaction was not significant (*p* = 0.541). Similarly, those who had irregular breakfast habits during the whole week consumed 28.8 g less fruit and vegetables compared with those that had breakfast every day (95% CI = −47.8, −5.2). The results were similar for boys and girls. After restricting the analyses to those that were classified as plausible energy reporters, the results were similar (results not shown). 

Girls with irregular breakfast habits on weekdays had on average 7.7 more sedentary minutes per day (95% CI = 0.8, 15.7) than girls with regular breakfast habits (Table 2). However, the association was in the opposite direction for boys, with those boys that had irregular breakfast habits having on average 13 min less sedentary minutes per day (95% CI = −25.3, −2.6) compared with boys that had regular breakfast habits. The interaction between breakfast habits on weekdays and gender was significant (*p* = 0.001) for sedentary time. Similarly, girls with irregular breakfast habits during the whole week also had 6.5 more minutes of sedentary time compared with girls with regular breakfast habits during the whole week (95% CI = 0.3, 14.1, interaction not significant *p* = 0.078). No significant associations were found between breakfast habits and MVPA.

A significant association was found between irregular breakfast during the whole week and higher levels of screen time on weekdays, with those that had irregular breakfast habits having around a 50% higher chance of having ≥2 h of screen time per day (95% CI = 1.1, 2.1), as shown in Table 3. Similar results were found for both girls and boys, although they were not significant for boys. 

## 4. Discussion

The results of this study show that irregular breakfast on weekdays was associated with lower intake of fruit and vegetables, more sedentary time among girls, and less sedentary time among boys. Interestingly, breakfast habits did not relate to MVPA. Additionally, skipping breakfast on at least one day of the whole week was associated with both lower fruit and vegetable intake and more screen time. 

Almost 40% of the participants skipped breakfast at least once during the week. The high prevalence of irregular breakfast habits is noteworthy, as such habits have been linked to lower academic achievements [26]. Another national Swedish study found that 13–17-year-olds reported eating breakfast 4.5–5.5 days a week, with those that came from families with higher education eating breakfast more frequently [27]. The proportion that skipped breakfast, reported by Wadolowska et al. (2019), was only 30%, although the participants were younger (11–13-year-olds) [16]. The proportion of 13-year-old participants that were irregular breakfast eaters in the study by Pedersen et al. (2012) was lower (ranging from 15–24%), though here, irregular breakfast consumption was defined as skipping breakfast on at least 2 weekdays [10]. As the definition of regular breakfast is not the same, it is difficult to draw any conclusions. 

The relation between screen time and skipping breakfast has been shown in a large sample of middle- and high-school students. High screen time was also found to be linked to low intake of fruits and vegetables and high intake of fast food [28]. However, no gender interaction was noted in that study. However, gender-specific dietary patterns have been identified in this age group previously [13]. Generally, girls have a higher Tanner score at a given chronological age, making it possible that these differences may be due to differences in maturity. In addition, variations in parenting between boys and girls may be of importance, although they were not assessed in this study. 

The mean fruit and vegetable intake in the current study was 219 g, which is similar (233 g) to a national survey among 11–17-year-olds where the same diet registration method was used [9]. Previous studies have shown that girls typically eat more vegetables and fruit than boys [9,29], but no significant differences were seen in our study. There was a significantly lower intake of fruits and vegetables among participants with irregular breakfast habits during weekdays but also when the whole week was considered. This has been seen in other studies as well. Pourrostami et al. found in a study from 2019 with children aged 7–18 years that skipping main meals such as breakfast, but also lunch and dinner, was associated with a lower frequency of fruit and vegetable intake [30]. 

One possible explanation for why the intake of fruit and vegetables was lower is that by foregoing breakfast, the adolescents miss out on the fruit and vegetables that would have been included in the breakfast. A previous study on younger children (7–10 years of age) showed that those with irregular breakfast habits also ate less fruit and vegetables on the days when they did eat breakfast [31]. This suggests that even if the breakfast would have been consumed, it might not have contained much fruit and vegetables among those with irregular breakfast habits. 

Boys had more than 15 min less sedentary time compared to girls as well as almost 5 more minutes in MVPA. This is in line with a previous Swedish study in a similar age group that showed that girls are more sedentary and spent less time in MVPA [13]. Our previously published study on this cohort showed differences in school-time MVPA but not in after-school leisure time MVPA [19], suggesting interventions in schools are important for increasing physical activity among girls. No differences were found between those with irregular and regular breakfast habits concerning MVPA. Several studies have also failed to find an association between breakfast habits and physical activity [32,33].

On the other hand, the association between breakfast habits on weekdays and sedentary time was significant but only after stratifying for gender. Interestingly, having regular breakfast habits on weekdays was associated with increased sedentary time in girls but decreased sedentary time among boys (interaction significant at *p* = 0.001). This suggests that the behavioral patterns regarding breakfast and sedentary behavior are different for boys and girls, and the reason for that is unclear. 

One previous study on the association between breakfast habits and sedentary time in a younger age group (9–11-year-olds) found no association between breakfast habits and overall sedentary time [34]. However, they found that having regular breakfast habits was associated with lower sedentary time in the morning [34], although the differences were quite small and were not stratified by gender. More commonly, previous studies have focused on screen time rather than accelerometer measured sedentary time. Although these studies found that irregular breakfast habits were associated with higher levels of screen time, they did not stratify by gender [16,17]. When we looked specifically at self-reported screen time, the results showed a 50% increased risk of having ≥2 h of screen time on weekdays for those with irregular breakfast habits during the whole week. The results were very similar in strength for boys and girls, although they were only significant for girls. The discordance in the results in the association between breakfast habits on weekdays and accelerometer measured sedentary time does not appear to be explained by screen time.

### Strengths and Limitations

The study has several strengths. The sample size was large with detailed measurements on intake of fruit and vegetables, device-measured physical activity and sedentary behavior as well as questionnaire data on breakfast habits and screen time for both weekend and weekdays. The dietary recall method has been validated previously in this age group [20] and allows for a high level of detail in the data. On the other hand, the dietary recall method was based on two days of dietary recall (and on only one day for 89 participants), which could have led to a bias in that estimate of the intake of fruit and vegetables. However, as the sample was large, these misclassifications should have evened out. Furthermore, sensitivity analyses were done on those that were classified as plausible energy reporters, which resulted in the same pattern of significant results. Asking the participants to register more days might have led to fatigue and drop-outs. Accelerometer-measured physical activity yields more objective and detailed information compared with self-reported measurements. The sample size was diverse, with about as many boys as girls and schools from both bigger and smaller municipalities, the city, and more rural areas. One limitation is the classification of regular and irregular breakfast habits. It could be argued that skipping breakfast once per week should not be considered as having irregular breakfast habits. Furthermore, the cross-sectional design of the study gives no information about causality. 

## 5. Conclusions

Sufficient intake of fruit and vegetables, being physically active, and limiting screen time are important factors for health and to reduce the risk of obesity, morbidity, and other risk factors in adults. Establishing these good habits early in life is favorable to continue to live a healthy life and therefore, it is necessary to investigate this in the young population. Since participants with regular breakfast habits had a higher fruit and vegetable intake and lower screen time, this knowledge is beneficial for promoting the importance of breakfast, to possibly increase the current low intake of fruit and vegetables. Additionally, the current study showed that one-third of the participants skipped breakfast on weekdays. Future research can continue to build upon this information to determine causal relationships and to develop interventions to increase fruit and vegetable intake as well as limit screen time.

## Figures and Tables

**Table 1:** Characteristics of the study sample.

	Total	Girls	Boys	
	*n* (%)	*n* (%)	*n* (%)	*p*
Number of participants	1139 (100)	580 (51.0)	558 (49.0)	
Age (mean ± SD. missing *n* = 0)	13.4 ± 0.3	13.4 ± 0.3	13.4 ± 0.4	0.147
Parental education (missing *n* = 169)				
≤12 years	275 (28.4)	144 (29)	131 (27.8)	0.674
>12 years	695 (71.6)	353 (71.0)	341 (72.2)	
Country of birth (missing *n* = 10)				
Sweden	967 (85.7)	490 (84.9)	476 (86.4)	0.758
Europe, including Nordic countries	46 (4.1)	24 (4.2)	22 (4.0)	
Outside Europe	116 (10.3)	63 (10.9)	53 (9.6)	
Municipality (missing *n* = 0)				
Rural	105 (9.2)	56 (9.7)	49 (8.8)	0.611
Urban	1034 (90.8)	524 (90.3)	509 (91.2)	
BMI categories ^1^ (missing *n* = 13)				
Underweight	89 (7.8)	38 (6.6)	51 (9.2)	0.203
Normal weight	815 (71.8)	430 (74.1)	384 (69.3)	
Overweight	179 (15.8)	89 (15.3)	90 (16.2)	
Obese	52 (4.6)	23 (4.0)	29 (5.2)	
Breakfast on weekdays (missing *n* = 8)				
Regular breakfast	768 (67.9)	367 (63.6)	400 (72.3)	**0.002**
Irregular breakfast	363 (32.1)	210 (36.4)	153 (27.7)	
Breakfast on weekends (missing *n* = 15)				
Regular breakfast	912 (81.2)	464 (80.6)	448 (81.9)	0.564
Irregular breakfast	212 (18.9)	112 (19.4)	99 (18.1)	
Breakfast for the whole week (missing *n* = 15)				
Regular breakfast	679 (60.4)	328 (56.9)	351 (64.2)	**0.013**
Irregular breakfast	445 (39.6)	248 (43.1)	196 (35.8)	
Screen time on weekdays (missing *n* = 13)				
≤2 h	360 (32.0)	179 (31.1)	180 (32.7)	0.566
>2 h	766 (68.0)	396 (68.9)	370 (67.3)	
Screen time on weekends (missing *n* = 16)				
≤2 h	178 (15.9)	70 (12.2)	108 (19.8)	**<0.001**
>2 h	945 (84.1)	506 (87.8)	438 (80.2)	
Fruit and vegetable intake (in grams) (mean ± SD, missing *n* = 6)	208.4 ± 174.3	205.5 ± 151.8	210.8±194.7	0.609
Sedentary time (in minutes) (mean ± SD, missing *n* = 236)	602.0 ± 66.6	608.9 ± 62.7	593.7±70.2	**0.001**
Moderate-to-vigorous physical activity (in minutes) (mean ± SD, missing *n* = 236)	52.0 ± 19.0	49.5 ± 17.7	54.9±20.1	**<0.001**

^1^ According to the International Obesity Task Force age-standardized cut-offs from 2012. Results in **bold** are significant at α < 0.05.

**Table 2:** Associations between breakfast (exposure), fruit and vegetable intake, sedentary time, and moderate-to-vigorous physical activity (outcomes).

	Fruit and Vegetable Intake (in Grams) ^1^		
	All	Girls	Boys
	**β (95% CI)**	**β (95% CI)**	**β (95% CI)**
Breakfast on weekdays			
Regular breakfast	REF	REF	REF
Irregular breakfast	**−26.65 (−49.31, −5.85)**	**−42.73 (−69.23, −14.57)**	−8.99 (−44.24, 24.90)
Breakfast on weekends			
Regular breakfast	REF	REF	REF
Irregular breakfast	−16.28 (−41.57, 9.74)	−1.44 (−34.45, 34.09)	−30.94 (−69.01, 10.42)
Breakfast for the whole week			
Regular breakfast	REF	REF	REF
Irregular breakfast	**−28.76 (−47.78, −5.22)**	−28.58 (−50.69, 3.62)	−28.87 (−59.00, 3.40)
	**Sedentary time (in minutes)** ^2^		
Breakfast on weekdays			
Regular breakfast	REF	REF	REF
Irregular breakfast	−1.36 (−8.01, 5.02)	**7.66 (0.75, 15.70)**	**−13.29 (−25.28, 2.56)**
Breakfast on weekends			
Regular breakfast	REF	REF	REF
Irregular breakfast	4.35 (−3.42, 12.68)	4.10 (−4.73, 13.63)	4.35 (−9.36, 18.65)
Breakfast for the whole week			
Regular breakfast	REF	REF	REF
Irregular breakfast	1.88 (−3.81, 8.20)	**6.49 (0.31, 14.14)**	−3.84 (−14.50, 7.16)
	**MVPA (in minutes)** ^2^		
Breakfast on weekdays			
Regular breakfast	REF	REF	REF
Irregular breakfast	0.62 (−2.13, 3.67)	−1.61 (−5.17, 2.34)	3.45 (−1.09, 8.22)
Breakfast on weekends			
Regular breakfast	REF	REF	REF
Irregular breakfast	−0.94 (−4.65, 2.13)	0.36 (−4.28, 4.82)	−2.27 (−7.76, 3.17)
Breakfast for the whole week			
Regular breakfast	REF	REF	REF
Irregular breakfast	−0.09 (−2.71, 2.63)	−0.47 (−4.05, 2.95)	0.26 (−4.18, 4.86)

^1^ Adjusted for gender, BMI (continuous), parental education, and energy intake. ^2^ Adjusted for gender, BMI (continuous), parental education, and accelerometer wear time. Results in **bold** are significant at α < 0.05. OR: Odds Ratio; CI: Confidence Interval; REF: Reference group; MVPA: Moderate-to-vigorous physical activity.

**Table 3:** Associations between breakfast (exposure) and high screen-time variables (outcomes).

	High Screen Time on Weekdays ^1^			High Screen Time on Weekends ^1^		
	All	Girls	Boys	All	Girls	Boys
	**OR (95% CI)**	**OR (95% CI)**	**OR (95% CI)**	**OR (95% CI)**	**OR (95% CI)**	**OR (95% CI)**
Breakfast on weekdays						
Regular breakfast	REF	REF	REF	-	-	-
Irregular breakfast	1.26 (0.91, 1.76)	1.39 (0.93, 2.06)	1.12 (0.71, 1.78)	-	-	-
Breakfast on weekends						
Regular breakfast	-	-	-	REF	REF	REF
Irregular breakfast	-	-	-	1.57 (0.91, 2.71)	1.57 (0.73, 3.38)	1.55 (0.75, 3.24)
Breakfast for the whole week						
Regular breakfast	REF	REF	REF	REF	REF	REF
Irregular breakfast	**1.53 (1.09, 2.13)**	B	1.49 (0.94, 2.37)	1.23 (0.74, 2.05)	1.17 (0.57, 2.39)	1.29 (0.77, 2.16)

^1^ Adjusted for gender, BMI (continuous), and parental education. Results in **bold** are significant at α < 0.05. OR: Odds Ratio; CI: Confidence Interval; REF: Reference group. - Only associations between the same type of days i.e., between breakfast on weekdays/whole week and screen time on weekdays were calculated.

## Data Availability

The datasets are not available for download in order to protect the confidentiality of the participants. The data are held at The Swedish School of Sport and Health Sciences.
